# Measuring Empathizing and Systemizing with a Large US Sample

**DOI:** 10.1371/journal.pone.0031661

**Published:** 2012-02-22

**Authors:** Daniel B. Wright, Elin M. Skagerberg

**Affiliations:** 1 Department of Psychology, Florida International University, Miami, Florida, United States of America; 2 Gender Identity Development Service, Tavistock and Portman NHS Foundation Trust, London, United Kingdom; The University of Queensland, Australia

## Abstract

A large number of people completed one of two versions of the empathizing quotient (EQ) and systemizing quotient (SQ). One version had the negatively phrased items all re-worded. These re-worded items were answered more rapidly than the original items, and for the SQ produced a more reliable scale. Subjects gave self-assessments of empathizing and systemizing, and these were moderately correlated, *r*≈.6, with their respective quotients. Females had on average higher empathizing scores and males had on average higher systemizing scores. If a female-male pair was chosen at random, the female would have the higher empathizing score about two-thirds of the time, and the males would have the higher systemizing score about two-thirds of the time.

## Introduction

Empathizing is the ability to identify with other people's thoughts and feelings and to be able to respond to these mental states with appropriate emotions [1]. Systemizing is being interested in understanding, predicting, and constructing rule-based systems. Several authors have described how lack of empathy can produce anti-social behavior in some people [2]. Lack of empathy is one of the characteristics often used to define antisocial personality disorders [3]. There is also discussion that difficulties with empathizing may be fundamental in autism [1]. During the past decade there has been much discussion, particularly of empathizing, because of how empathetic processes that might build on collections of mirror neurons affect social cognition [e.g.], [ 4,5]. This would predict that people with autism would have deficits with mirror neuron systems [6]. While many studies have found this [7], others have not [8].

Baron-Cohen describes the systemizing mechanism which “drives the brain to look for input-operation-output relationships in any data, and to construct systems” [9, p. 868]. People vary in how high this drive is, and people with high systemizing drive also prefer systems with high regularity (e.g., calendars) rather than systems with more erratic behavior (e.g., people). Baron-Cohen describes one way in which empathizing and systemizing relate. Both can be used to try to predict the behavior in others. Empathizing (and even anthropomorphizing for non-humans) can be used by attributing emotions, goals, etc., like our own to others. Systemizing can be used by constructing rules for the others and predicting behavior based on these rules. If someone has difficulty with either of these, the person could use the other to compensate (although each method will not be well suited for some tasks).

The concern of this paper is how empathizing and systemizing can be measured. Two of the most used scales for measuring empathizing and systemizing are the empathizing quotient (EQ) and the systemizing quotient (SQ) developed by Baron-Cohen, Wheelwright and colleagues at University of Cambridge [10], [11]. Methodological work on the factor structure of these scales has been conducted [12], [13].

**Figure 1:**
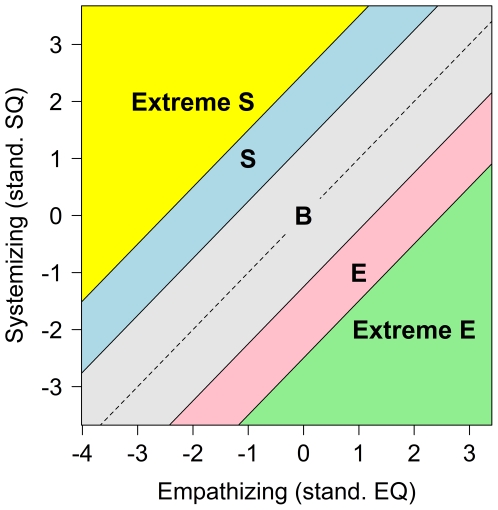
Using the difference between EQ and SQ for classifying cases.

When plotting the results from the EQ and SQ, Baron-Cohen and colleagues claim five different ‘brain types’ can be described [14]. These are depicted in Figure 1. To create this classification scheme, first, both variables need to be standardized (for the non-clinical group if there are multiple groups) so that the standardized scores have a mean of zero and a standard deviation of one. Once the difference between these standardized quotients is calculated, the bands can be calculated in standard deviation units of this difference. Different papers use different methods to calculate the width of these bands. In Figure 1 the grey band, labeled B for Balanced, is for people who have a difference of less than one standard deviation from 0. People who have a difference score over one standard deviation are classified as either S (for Systemizing in light blue) or E (for Empathizing in pink), depending on which quotient is larger. If their difference is greater than two standard deviations then they are Extreme S, in yellow, or Extreme E, in green.

Females score higher on average on EQ and therefore are often classified as type E and males score higher on average on SQ and therefore are often classified as type S, prompting Baron-Cohen to argue that these constructs underlie what might be thought of as the prototypical female and male brain types [1]. Additionally, individuals with Autism Spectrum Conditions (ASC) tend to score higher than typical males on systemizing and lower than typical males on empathizing, suggesting that people with these conditions show an “extreme male brain profile” [15]. The notion of ASC being a form of extreme *maleness* goes back over 70 years to Asperger [16], [ as cited in 17].

According to Figure 1, it is the imbalance of the empathizing score and the systemizing score that is critical for classifying types. This is based in part on Baron-Cohen's argument that people with low ability for empathizing or systemizing may use the other to compensate [9]. He describes how people with ASC, who have difficulty empathizing, can become hyper-systemizers. While this compensation aspect may be very important when considering groups with particularly low values on one of the other of the constructs, it may be less important considering the general population. It may be that having extreme empathy (low or high) or extreme systemizing (low or high) is more important than the balance between the two constructs. The Extreme S and S groups from Figure 1 could include people with average or below average scores on SQ if their EQ scores are low enough. Similarly Extreme E and E groups could include people with average or below average scores on EQ if their SQ scores are low enough. These situations do occur for our data set. There are numerous other ways in which people could be categorized by their scores on these two measures. If the EQ and SQ each measure important meaningful constructs, then it is worth exploring categorization schemes that are based on each on its own rather than on the difference between them. The one in the left panel, called Alternative 1, labels people who are within one standard deviation as “average” (this could be increased to two or more standard deviations if researchers want to focus on more extreme cases). People can then be classified as having high S and low E (yellow), high E and low S (green), high E and high S (pink), and low E and low S (blue). Alternative 2, in the right panel, adds further classifications for those who score high on one measure, but are within one standard deviation of the mean on the other. Alternative 2 requires more categories, but loses less information when classifying cases. It also treats the measures as completely independent. For both of the graphs the axes are in units for the means of the scales.

**Figure 2:**
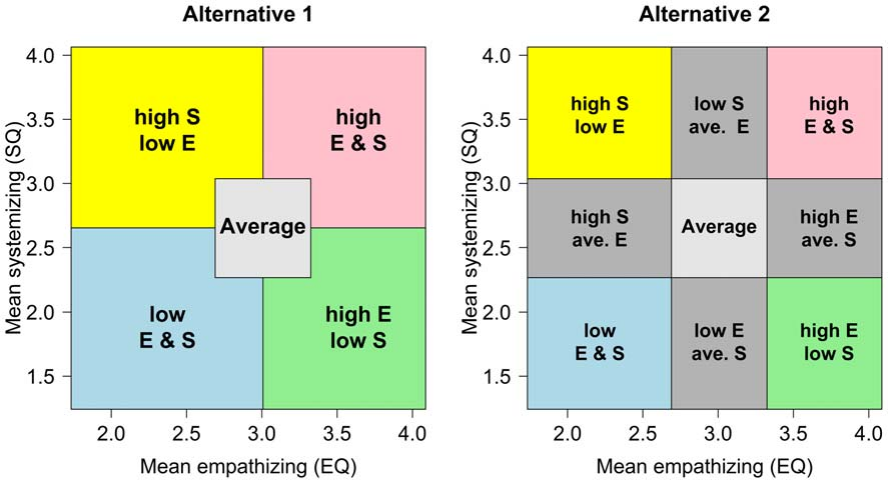
Classifying cases for EQ and SQ treating them as independent. Alternative 2 is more complex, but differentiates people scoring near the average from those scoring more extreme.

The important difference between Figures 1 and 2 is how people with similar but extreme scores of EQ and SQ are treated. Within Figure 1 they would be classified equivalently, while within both schemes shown in Figure 2 they would be classified differently. The choice of which scheme of these should be preferred, or whether another scheme should be used, will be based on the researchers' needs. All three of these classification schemes could be useful in some circumstances so we report normative data for all three schemes for our data. Further, in many circumstances it is best to report individual standardized scores rather than classify the person.

### Current Study

Our interests are threefold. First, there are some items on both the EQ and the SQ which are syntactically complex due to having negative phrasing. For example,

*When I learn about historical events, I do not focus on exact dates.*

*I do not find it distressing if people who live with me upset my routines.*

Processing complex phrases (i.e., saying *not do* something) is often more difficult than processing simpler phrases (i.e., saying *do* something) and this can lower the reliability of the responses [18]. Further, if the phrases are changed to positively worded questions, they tend to use fewer words and therefore can be processed more rapidly. For example:

*When I learn about historical events, I focus on exact dates.*

*I find it distressing if people who live with me upset my routines.*

Each of these two items has two fewer words than the original items. The main reason why questionnaire developers often phrase items negatively is to take into account that some people may tend to give responses on one end of the scale. This allows researchers to have approximately the same number of questions that require high answers for both ends of the psychological construct. We developed alternative versions of these questionnaires. For five of the EQ items the negative phrasing was changed to positive phrasing and for 28 of the SQ items the negative phrasing was changed. We predict that response times will be faster for the positively phrased items than for the negatively phrased items. We expect that if there is a difference in the reliability between the two versions, the new version will be better because of the difficulties people have processing complex questions in surveys (Schuman & Presser, 1981).

Second, we explore how accurate people are at judging their own levels of empathizing and systemizing. Besides administering the EQ and SQ, subjects were given definitions of these constructs and asked to make self-assessments. We believe that there will be positive associations between the scores from the psychometric scales and the corresponding self-assessments.

Finally, we wanted to gather normative data, particularly in relation to gender differences, from a large US sample. Much research on these measures has been done in other countries, but with sampling procedures that are different from ours. The group at Cambridge has accumulated a large amount of data (over 5000 people) through the Autism Research Centre website in the UK. [12]. Wakabayashi and colleagues collected data from ASC individuals, students, and employees in some companies in Japan [19]. The employees group was relatively small (*n* = 137) and no details of how the companies were sampled was provided. Both these studies found that females had higher EQ scores than males, but males had higher SQ scores. Studies with students also find these gender differences [e.g., 20]. All sampling methods will have biases. One aim of the current study was to have a large sample not self-selected because of their interests in either autism or in these scales. This will allow us to estimate the size of the gender differences on these scales. We predict that females will have higher mean empathizing scores and lower systemizing scores, but we expect the distributions to overlap. We provide estimates of these associations which communicate the size of the effect (and the overlap) in terms that are easily communicated to non-scientific audiences. We also compare these scores with scores on a scale for values along the autistic spectrum, the AQ [21]. Given the sample size, we can also estimate the proportions of people in the different classifications depicted in Figures 1 and 2.

## Methods

### Ethics Statement

The research reported here was conducted in accordance with the ethical guidelines of the American Psychological Association. Informed consent was received from each participant via the computer (each ticked a button to say they read the consent form and agreed to take part). This form was included in the proposal to the university's ethics board. The research was given official clearance by the Florida International University Institutional Review Board.

### Participants

The online survey company, *Qualtrics* (www.qualtrics.com), administered our survey. They contacted a subset of their pool of four million people and asked these people to take part in the study. Members of their pool receive compensation from Qualtrics based on the number of surveys that they complete.

To help to determine the sample size, a power analysis was conducted. Because the SQ has a high Cronbach's α, around .9 [21], being able to detect an increase is likely to require a large sample. Bonett provides power equations for comparing two αs [22]. The minimum effect that it was felt would be worth detecting is a shift of .020. To do this with a power of 95% and a critical *p* value of 1% requires approximately *n* = 1350 per condition. Therefore, it was necessary to have approximately 1500 participants per condition to finish the survey. As is regular practice for online studies, more people than necessary are included in the sample because some do not complete the survey and others do not spend sufficient time on questions or do not follow instructions. Filter questions were interspersed throughout the survey instructing subjects to respond in a particular way. These questions help to ensure that subjects are reading the items correctly.

Eight hundred and nine subjects answered at least one of the filter questions in a way other than how they were told, so were excluded from the analyses. In total, 5186 completed all the items on both the EQ and the SQ (2597 the unchanged versions, and 2589 the changed versions) and answered all the filter questions as instructed. An additional 228 completed the EQ, but failed to complete all the items of the SQ. Participants filled out basic demographic questions including whether they were male or female, and their age from the following responses: 18–24, 25–34, 35–44, 45–54, 55–64, 65–74, 75–84, 85+ years old. In accordance with university ethics guidelines, subjects were not required to answer the demographics questions. More than 99% answered these questions. Of those who answered, 41% were male and the median age was in the 45–54 years of age bracket.

### Questionnaires

The original EQ had 60 items, but only 40 of these were used for scoring. The version currently recommended on the Cambridge website uses these 40. The original SQ also has been revised. More of the systems referred to in the original version were traditional male systems. This has been rectified in the SQ-R. In addition subjects filled out the Autism Spectrum Quotient (AQ) [21]. A shortened version of the AQ was used so that the full study could fit into the time allotted for the survey [23]. This is not the focus of the current report, but it is discussed briefly in the results. The three questionnaires were presented in this order for every subject.

For each item, the statement would appear on the screen with four response alternatives: strongly agree, agree, disagree, and strongly disagree. We code these responses as 1–4. Sometimes these scores are treated as binary variables: combining strongly agree and agree, and combining disagree and strongly disagree, and sometimes as 4-point rating scales. Here they are treated as 4-point rating scales. Further, sometimes an individual's EQ and SQ are calculated by summing the number of responses in the direction of the construct. Because the different versions of these questionnaires have different numbers of items, this can create difficulties comparing across versions (which should always be done cautiously). Therefore, the means of items are reported rather than their sums. The software recorded the time between the item appearing on the screen and the subject's response.

Two versions of each questionnaire were made. The difference was whether the negatively worded items were made positive. This occurred for five of the EQ items, 28 of the SQ items, and a single AQ item. The Qualtrics software randomly allocated subjects to either the original versions or those with the negatively worded items changed. A subject either received all the items in the original form, or had all the 34 critical items altered. In addition, the questionnaires were changed so that they had American spellings and words (e.g., *motorway* was changed to *highway*).

After filling out the questionnaires the participants were given the following descriptions of empathizing and systemizing:

Empathizing is about being able to identify with other people's thoughts, emotions, and feelings. Some people empathize with other people naturally, without much effort. Other people are less able to empathize with the thoughts, emotions, and feelings of others.

and

Systemizing is about being interested in how systems work. By system we mean anything that has some structure and follows a set of rules. Systems can be physical machines, like a radio, biological systems, like your own body, or social systems, like how groups of people behave. Some people are very interested in understanding the structures and rules of different systems, other people are less interested.

They were given a scale with a bar that they could move with their mouse and asked how much each of these constructs applied to them compared with others.

A debriefing page thanked the subjects for taking part. The study took approximately 20–25 minutes.

### Methods of Analyses

The sample is self-selected both to be part of the Qualtrics panel and to be in the study (see http://www.websm.org/ for archive of web survey research related to sample coverage). At minimum, all subjects will have access to, and experience with, computers. For the statistical analyses, where applicable the effect sizes are all reported in units of the original variables. Where means are compared, ANOVAs and *t*-tests are reported. Where associations are estimated, Pearson correlations are reported. Questionnaire reliability is measured with Cronbach's α.

## Results and Discussion

The results are divided into three sections. First, we examine the results for the methodological research question on whether changing the negatively worded items to positively worded items affects the response times and reliability. Second, we examine how closely the scores on the EQ and SQ relate to people's self-assessments of having these traits. Third, we report variability of these scores for gender, age, and scores on the AQ. Items were reversed scored where appropriate and unless otherwise specified all means are on a 1–4 scale.

### Are Negatively Worded Items Good or Bad?

**Figure 3:**
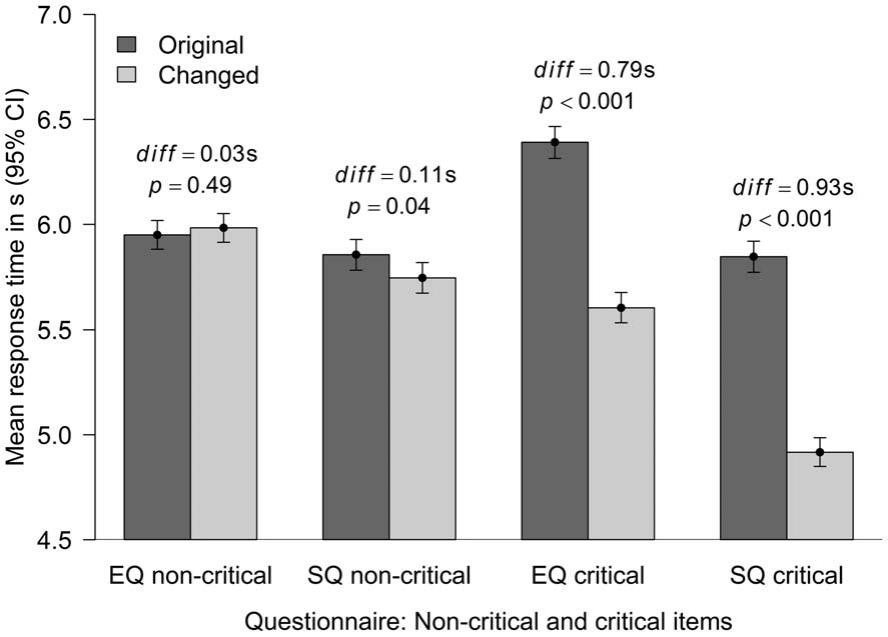
The mean response times for the non-critical items for EQ and SQ (which were the same items) and for the critical items for the EQ and SQ (where the original versions were negatively phrased and the changed were not).

It was predicted that the subjects would be faster responding to the changed version items. Response times that were less than .2 s were set to .2 s and response times greater than 10 s were set to 10 s. The mean response times for the five critical EQ items and the 28 critical SQ items were calculated. We also found the means for the remaining non-critical items, but predicted that any differences between these for the two versions would be small. Figure 3 shows the means and 95% confidence intervals for these response times for the two versions. The response times for the non-critical items are similar. For the EQ the difference is not significantly different from zero. For SQ, the *t* test reached the traditional level of significance, *t*(5164) = 2.10, *p* = .04, but the effect is small. For the critical items the response times for the changed version are between 3/4 and 1 second faster than the original version: *t*(5403) = 14.66, *p*<.001, for the EQ; *t*(5172) = 18.19, *p*<.001 for the SQ. Only a single item was changed for the AQ (“It does not upset me if my daily routine is disturbed” to “It upsets me if my daily routine is disturbed”). The new version was answered 0.90 s faster, 4.27 s versus 5.17 s, which is statistically significant, *t*(5136) = 12.67, *p*<.001. The means for the remaining items of the AQ were similar for the two groups, 4.74 s for the changed group, 4.75 s for the original group, *t*(5166) = 0.19, *p* = .85.

**Figure 4:**
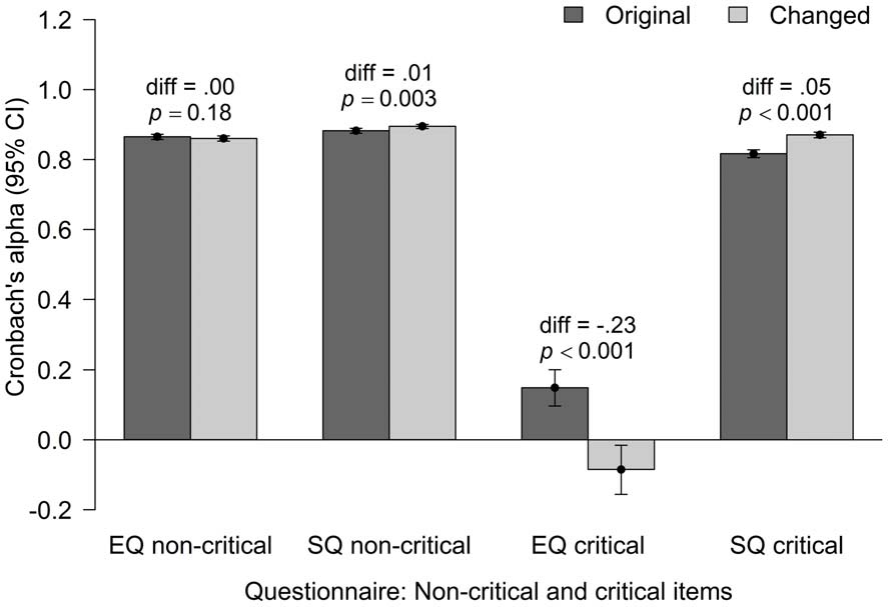
The Cronbach α values for the non-critical items for EQ and SQ (which were the same items) and for the critical items for the EQ and SQ (where the original versions were negatively phrased and the changed versions were positively phrased).

Next we examined how well the critical items fit together using the unstandardized Cronbach's α on the five critical items for EQ and the 28 critical items for SQ. The α values and their 95% confidence intervals were calculated using the cronbach.alpha function [24]. Statistical tests of the differences between the α values were done using the procedure described in Feldt and Kim [25]. Figure 4 shows the values for the original unchanged items, for which we expect no differences, and for the critical changed items. Because there are a large number of non-critical items, their confidence intervals are small. The difference for the non-critical items of the SQ was statistically significant, despite the items being the same. This is likely to be caused by a residual effect of these subjects having had to process the more difficult critical items. This might also account for why the response times were slightly slower for these items.

The differences were greater for the critical items. Because there are only five critical items for the EQ (compared with 28 for the SQ), the confidence intervals are much wider than for the SQ. The new version of the EQ had *lower* reliability than the original version. Importantly, both of these versions had low reliability. Examination of the correlation matrices showed that half of the 20 correlations were negative, meaning that these items do not covary in the intended manner. The effect for the SQ items is statistically significant, *F*(2504,2495) = 1.42, *p*<.001, in the predicted direction, with α increasing from .82 to .87. Cronbach αs were calculated for the different versions for all the items. For the EQ the values were similar: .866 (95% CI from .859 to .873) for the unchanged version; .857 (95% CI from .849 to .865) for the changed version; difference *F*(2655,2619) = 1.065, *p* = .05. The reliabilities for SQ were from .913 (95% CI from .907 to .918) for the unchanged version to .937 (95% CI from .933 to .941) for the changed version; difference *F*(2562,2554) = 1.396, *p*<.001.

The relationship between the EQ scores on the critical items and the non-critical items were examined for both the original and the change versions. The scores were calculated by taking the mean response for each set, after reverse scoring where necessary. This was done also for the SQ. The correlations are shown in Table 1. The correlation for the changed EQ was significantly lower than the value for the original EQ, *z* = −2.80, *p* = .005. The correlation for the changed SQ is significantly higher than for the original SQ, *z* = 18.95, *p*<.001. The correlation between the single AQ critical item and the remainder of the scale was slightly, but non-significantly, higher for the changed version, *z* = 1.90, *p* = .06.

**Table 1:** The Pearson correlations, and their 95% confidence intervals in parentheses, between the scores on the critical items and the remaining items for the unchanged and changed versions, for EQ, SQ, and AQ.

	Original	Changed
EQ	.477 (.447, .505)	.416 (.383, .446)
SQ	.650 (.630, .671)	.862 (.852, .872)
AQ	.284 (.249, .319)	.332 (.298, .366)

In summary, as predicted the response times were faster for the items that had been changed. The difference was between half of a second and one second. The changed items were less complex and therefore should have been easier to understand. However, for the EQ the reliability of these new items and the correlation between them and the remainder of the scale were lower for the changed version than the original version. Thus, while we encourage further development and testing of the EQ, we do not recommend changing the negative phrasing of these five items to positive phrasing.

For the SQ the response times were also faster for the changed version than for the original. Further, the internal reliability of those items was higher, and the correlation between these items and the remainder of the scale was higher for the changed version than for the original version. There even appeared to be some residual effect on the processing of the unaltered items, in terms of both response times and reliability, because of the difficulty of the negatively phrased SQ items. Thus, we do recommend that researchers consider changing the phrasing of items on the SQ from negative to positive. Changing the single AQ item resulted in quicker responses, but on the basis of a single item we make no recommendations.

### Can People Assess if they are Empathizers or Systemizers?

**Figure 5:**
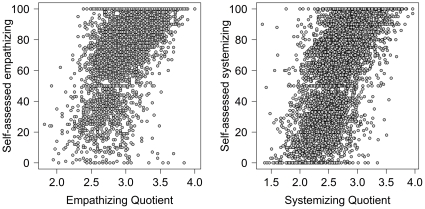
Scatter plots comparing people's self-assessed ratings of empathizing and systemizing with their scores on the EQ and SQ. Empathizing scores are shown in the left panel, systemizing scores in the right panel.

Figure 5 shows the relationships between EQ and SQ with subjects' self-assessed beliefs about their levels of empathizing (left panel) and systemizing (right panel). Subjects' beliefs are positively associated with the scores from the corresponding psychometric measure. The Pearson correlations were: *r* = .591 (95% CI from .573 to .609) for empathizing and *r* = .595 (95% CI from .576 to .612) for systemizing. It is worth noting that the correlations between the other potential pairings were much smaller. The two self-assessments were correlated *r* = .136 (95% CI from .109 to .163). The EQ and SQ were correlated *r* = .219 (95% CI from .193 to .244). The self-assessed empathizing was correlated *r* = .170 (95% CI from .143 to .196) with SQ, and the self-assessed systemizing was correlated *r* = .045 (95% CI from .017 to .072) with EQ. It is worth stressing that correlations are greatly affected by extreme groups. Thus, if an ASC group was included, their scores would likely be much higher for systemizing than empathizing, and this would lower the EQ-SQ correlation. The positive correlation found here between EQ and SQ differs from the week negative correlation found in Wheelwright et al. [21] Differences in sampling are the likely explanation.

When examining the distributions of the self-assessments, many subjects gave ratings at 0 (1.3%), 50 (2.2%), and 100 (9.7%). Tobit regressions were conducted treating these as doubly-censored data. The general interpretation was the same; that in Cohen's terms there are medium sized correlations between the self-assessments and the psychometric measures [26].

In addition we examined if the correlations were similar for males and females. They were. The correlations for self-assessed empathizing with EQ were: .59 (95% CI from .56 to .62) for males and .55 (95% CI from .52 to .57) for females. The correlations for self-assessed systemizing with SQ were: .56 (95% CI from .53 to .59) for males and .58 (95% CI from .55 to .60) for females.

### Exploring EQ and SQ Differences by Gender, Age, and AQ Score

The most discussed demographic differences for EQ and SQ are by gender [1]. As predicted, in our sample females scored higher on empathizing than males: 3.089 versus 2.897, *t*(5070) = 22.35, *p*<.001, and males scored higher on systemizing than females: 2.757 versus 2.582, *t*(5070) = 16.29, *p*<.001. The standard deviations of these measures are: EQ for males .309, EQ for females .297, SQ for males .376, and SQ for females .374. Thus, the gender difference for EQ is .63 of a standard deviation and for SQ is .47 of a standard deviation. In Cohen's terms, these are medium sized effects [26].

**Figure 6:**
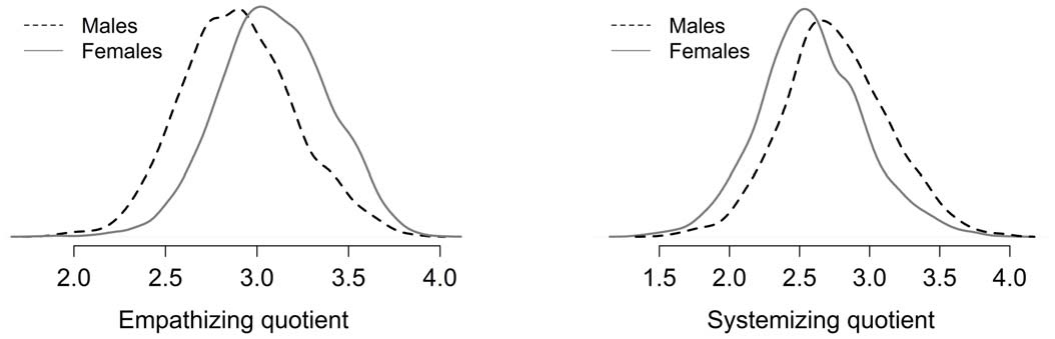
The distribution of mean responses for males and females for the EQ (left panel) and the SQ (right panel). The distributions are drawn using a Gaussian kernel estimation method (the R default).

Most people do not think in terms of shifts in standard deviations. Figure 6 shows the distributions for the two measures. While the gender differences are of a medium size (in Cohen's terms) and statistically significant, the distributions overlap. If one male and one female were sampled at random from these distributions (these percentages are based on sampling a million pairs), 66.76% of the time the female would have the higher empathizing score, 31.11% of the time the male would have the higher empathizing score, and 2.13% of the time the scores would be identical. For the systemizing scores, the males would have the higher score 62.72% of the time, the female the higher score 36.33% of the time, and they would have the same score 0.95% of the time. An R function for calculating this statistic is shown in Appendix S1.

**Figure 7:**
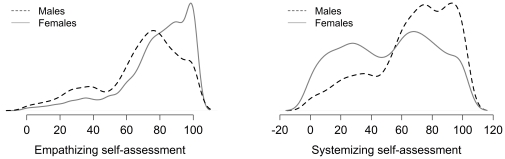
The distribution of mean responses for males and females for the self-assessed empathy ratings (left panel) and the self-assessed systemizing ratings (right panel). The distributions are drawn using a Gaussian kernel estimation method (the R default).

We also tested whether the scores on the self-assessed empathizing and systemizing questions varied by gender. Figure 7 shows the distributions for these. The distributions are not as smooth as those for the EQ and SQ, but given that they are based on only a single question it is reassuring that they do show the same basic finding. Females have a higher mean empathizing score than males, 77.97 versus 68.16, *t*(5070) = 15.36, *p*<.001, shift of .44 of a standard deviation. Males have a higher mean systemizing score than females, 67.16 versus 51.90, *t*(5070) = 18.95, *p*<.001, shift of .55 of a standard deviation. As with the psychometric scores, we randomly choose one million male-female pairs. For empathizing, females were higher on 62.03%, males were higher on 35.19%, and the pair had the same score on 2.78%. For systemizing, males were higher 64.24%, females were higher 34.13%, and the pair had the same score on 1.62%.

**Figure 8:**
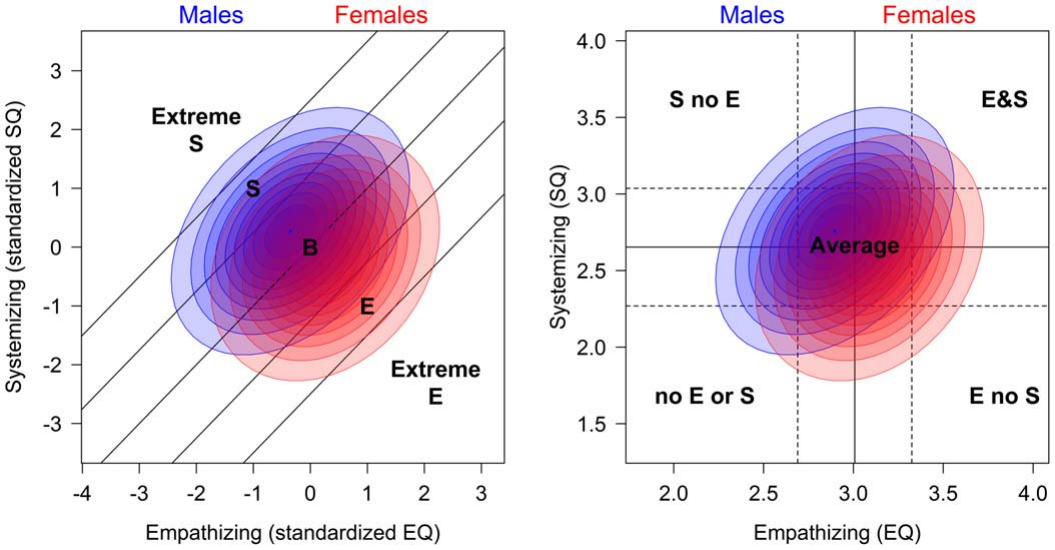
Gender differences (assuming bivariate normality) for the classification schemes shown in **Figures 1** and **2**. Each band in these figures represents 10% of males and females. The outer ellipse includes 90% of the population for males (in blue) and females (in red).

As discussed in the introduction, Baron-Cohen combines scores on the EQ and the SQ by taking the difference between them and uses this difference to classify people (Figure 1) [1]. This is one of several possible classifications. The right panel of Figure 2 shows a classification scheme that treats the constructs measured by EQ and SQ as independent. Figure 8 shows the gender differences superimposed onto the classification schemes of Figure 1 and the right panel of Figure 2. The ellipses show approximately where the data for males and females lie. The outer ellipse includes 90% of the data, the next ellipse 80% of the data, and so on. Tables 2,3,4 give the proportion of males and females in each group according to each classification scheme. The schemes each show gender differences where predicted. However, the classification scheme based on Baron-Cohen and colleagues, shown in the left panel, does classify a small percentage of people with Extreme Systemizing when they score below the mean for SQ (3%), and a small percentage with Extreme Empathizing who score below the mean for EQ (1%). Seventeen percent of people classified as having Brain Type S had SQ scores below the mean and 13% of people classified as having Brain Type E had EQ scores below the mean. The percentages found here differ from those found by the Cambridge group [27], [21]. The classification methods are different. In the top part of their Table 1 classification is based on differences in the medians for males and females, and on scores for an ASC group. In the lower part it is based on percentiles. The basic conclusion, that males score higher on systemizing and lower on empathizing than females, is found in both studies.

**Table 2:** The proportion of males and females by the classification scheme shown in Figure 1.

	Extreme S	S	Balance	E	Extreme E
Male	3.3%	23.3%	68.6%	4.2%	0.6%
Female	0.7%	5.1%	71.1%	18.8%	4.3%
Total	1.8%	12.5%	70.0%	12.9%	2.8%

**Table 3:** The proportion of males and females by the classification scheme shown in the left panel of Figure 2.

	S no E	No S no E	Average	S & E	E no S
Male	16.5%	18.3%	45.8%	16.3%	3.0%
Female	3.6%	13.5%	50.5%	16.9%	15.5%
Total	8.9%	15.5%	48.4%	16.7%	10.6%

**Table 4:** The proportion of males and females by the classification scheme shown in the right panel of Figure 2.

	Male			Female		
	Low S	Medium S	High S	Low S	Medium S	High S
Low E	3.9%	15.7%	1.9%	2.3%	5.3%	0.5%
Medium E	4.8%	45.8%	15.7%	13.3%	50.5%	5.5%
High E	0.6%	3.6%	5.3%	3.4%	14.1%	5.0%

Any classification scheme which combines scores on two variables is going to produce anomalies like this compared with other schemes. Because labels often can be mis-interpreted and often can distance people from the actual measurements on which they are based, it is important to make sure the labels accurately describe how the classification is made. If the important aspects of being an empathizer or a systemizer are measured by differences between EQ and SQ, then the scheme of Figure 1 should be preferred. If the important aspects of being an empathizer are measured by EQ and the important aspects of being a systemizer are measured by SQ, then the alternative schemes of Figure 2 should be preferred. Alternative 2 is the more complex of these, but may be useful because arguably people within one standard deviation of the mean should not be labeled as empathizers or systemizers.

**Figure 9:**
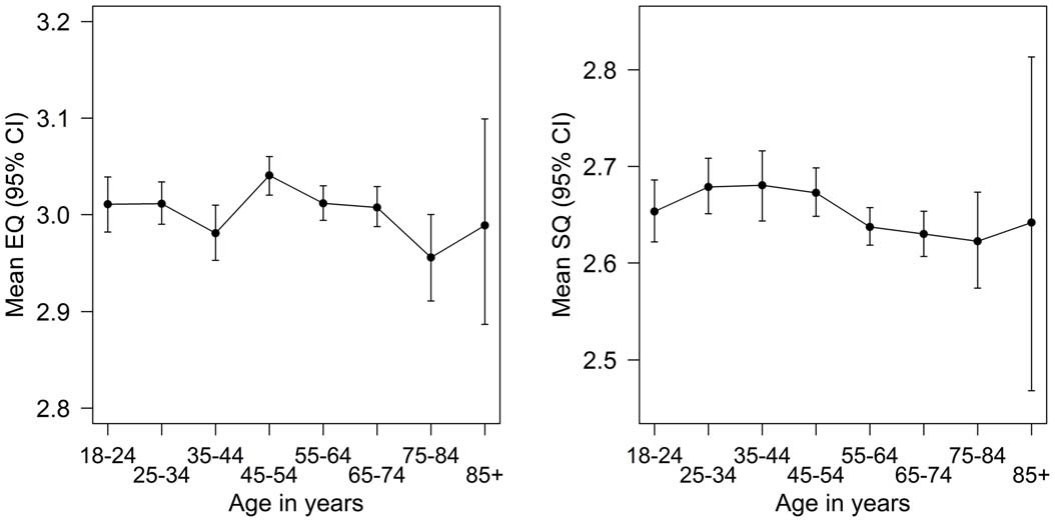
The mean EQ (left panel) and SQ (right panel) scores for different age bands. The means could potential range from 1–4 so these graphs show only a small part of this range.

Figure 9 shows the mean EQ and SQ scores for the different age bands. The means could potentially range from 1 to 4, so only a small amount of this potential range is shown. For EQ, while the one-way ANOVA treating age as 8 categories is statistically significant, *F*(7,5050) = 2.84, *p* = .006, the effect size is small, η^2^ = .004. Further, the effect does not show any clear pattern, *r* = −.01, 95% CI from −.04 to .02, *p* = .49. For SQ, the ANOVA just reaches the traditional level of significance, *F*(7,5050) = 2.12, *p* = .04, but the effect is small, η^2^ = .003. There does appear to be a possible non-linear pattern in this graph, but adding neither the quadratic term nor the cubic term fit significantly better than the linear model. Given that the mean scores are all between 2.62 and 2.69, the main conclusions for the relationship between the EQ and the SQ with age are that any differences which exist are small.

**Figure 10:**
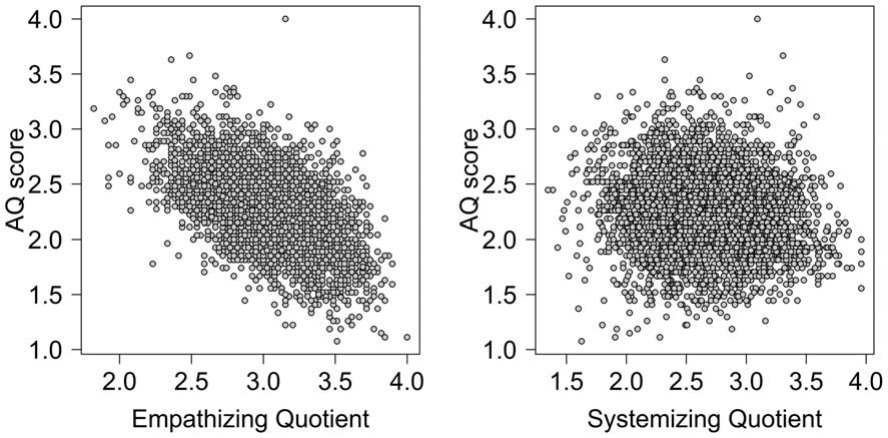
Scores on the AQ with scores on the EQ (left panel) and SQ (right panel).

Next, EQ and SQ scores were compared with AQ scores. Figure 10 shows that EQ has a moderate to strong negative correlation with AQ, *r* = −.58, 95% CI from −.60 to −.56, *t*(5181) = 51.57, *p*<.001, while SQ has only a small negative correlation with AQ, *r* = −.11, 95% CI from −.14 to −.08, *t*(5181) = 7.92, *p*<.001. The negative correlation between EQ and AQ is consistent with other findings [21]. However, in their study they found a moderate positive correlation between SQ and AQ which was not observed with our sample.

**Figure 11:**
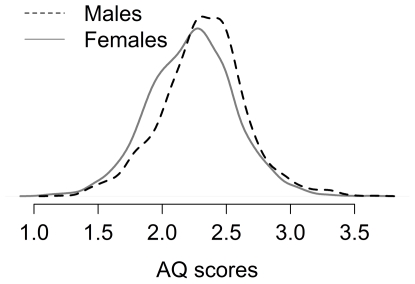
The distribution of mean responses for males and females for the AQ. The distributions are drawn using a Gaussian kernel estimation method (the R default).

There was a relationship between AQ scores and gender. Males had a mean of 2.32 (sd = .33) while females had a mean of 2.23 (sd = .33). The difference is statistically significant, *t*(5070) = 9.65, *p*<.001, but is only about one-quarter of a standard deviation (see Figure 11). If one male and one female were chosen at random from this sample, 56.44% of the time the male would have the higher AQ score, 40.34% of the time the female would have the higher AQ score, and 3.22% of the time they would have the same score. While treating the age bands as categorical yielded a statistically significant result, *F*(7,5050) = 2.32, *p* = .02, η^2^ = .003, the effect was very small and there was no discernible pattern in the results, *r* = .02, 95% CI from −.01 to .05, *t*(5056) = 1.36, *p* = .17.

### Summary

Empathizing and systemizing are important constructs for how we interact with other people based on their emotive states and desires and for how we interact with the wide variety of systems encountered daily. The focus of this paper is on how they are measured and establishing normative data. Baron-Cohen has described people with extreme levels of these constructs [1], [2], [9]–[11], [14], [15]. He has developed the notion of different brain types for empathizers and systemizers, and has shown that females are more likely to be empathizers and males more likely to be systemizers. Using a large non-student sample and using his classification method, we found 5% of males were empathizers (either E or Extreme E) compared with 23% of females, and 27% of males were systemizers (either S or Extreme S) compared with 6% of females. It is important to stress, as Baron-Cohen does, that this association does not mean that all males differ on these constructs from all females. As evident in Figures 6,7,8 there is a large overlap.

The classification scheme depicted in Figure 1 focuses on the difference between scores on the EQ and SQ. This classification scheme is particularly diagnostic for ASC because people with ASC tend to have much lower EQ scores than SQ scores. The interplay between these measures is important. Social cognition research describes how empathizing is an important skill for dealing with social systems. It may be that people who are poor empathizers, but good systemizers, may develop skills for dealing with social systems that rely less on empathy [1], [9]. Longitudinal research on these constructs would be welcome.

In order to continue theorizing about these constructs it is critical to continue to improve the measurement instruments. This study had included a methodology experiment. Half of the people responded to the EQ and SQ as currently recommended on the Cambridge website; half had the negatively phrased items changed so that they were positively phrased. This simplified the questions as shown by response latencies. It did not improve the reliability of the EQ, but closer examination of the critical items showed that neither the original nor the changed items correlated together well. We recommend closer examination of these items. It did improve the reliability of the SQ. Therefore we recommend changing the negative phrasing of these items to be positively phrased.

## Supporting Information

Appendix S1 **A distribution-free and ordinal measure of effect size.**
(DOCX)
