# Supplementary material for: Measuring Empathizing and Systemizing with a Large US Sample
Source: PLoS One. 2012 Feb 22;7(2):e31661. doi: 10.1371/journal.pone.0031661 (PMC3285168; doi:10.1371/journal.pone.0031661)
Supplement: Appendix S1 — A distribution-free and ordinal measure of effect size. (DOCX) [file pone.0031661.s001.docx]

**S1. Appendix: A distribution-free and ordinal measure of effect size**

There is much debate about different effect size measures for comparing two groups on a scale. Most of the standardized measures are based on differences in the means divided by an estimate of the standard deviation, on proportion of "variance accounted for", or on overlap between distributions. These notions can be difficult to convey the size of the effect to non-statisticians. An intuitive measure is if you chose a person, at random, from each group how often is the person from one group going to have the higher value. Most of the effect sizes mentioned above make assumptions about the distributions of the responses for the two groups. Assuming the distribution for each group is normal with means of μ1 and μ2, and standard deviations of σ1 and σ2, then the difference between two people, each randomly chosen from one of the groups, is normally distributed with a mean μ1 - μ2 and standard deviation of $\sqrt{{\sigma1}^{2}+{\sigma2}^{2}}$. The probability of observing any pair with one group being larger or smaller can be found by plugging in the sample values and using tables of the normal distribution or the statistic functions which are part of all major statistics packages.

For EQ, the mean for males was 2.90 and the mean for females as 3.09, with standard deviations of .31 and .30 respectively. The distribution for the difference is estimated to have a mean of .19 and a standard deviation of .43. Using these values shows that 67% of the time it is expected that the female will have the higher score and 33% of the time the male will have the higher score. This approach requires both making distributional assumptions and using (albeit a small amount of) mathematics related to the distributions. To make the effect size as intuitive as possible it was important to rely on as little mathematics as possible. The solution which we felt required the least mathematics was to choose at random one male and one female, and record which had the higher value (or if it was tied), and let the computer repeat this task over and over again.

R is the fastest growing statistics resource and it is free. Therefore, a function to calculate this statistic was written in R (details of downloading R are available at www.r-project.org). The function, called paircompare, is:

paircompare <- function(x,y,reps=10000,norm=FALSE){

ok <- complete.cases(x,y)

x <- x[ok]; y <- y[ok]

if (length(unique(y)) != 2)

stop("Second argument must have only 2 valid values")

if (norm){

means <- tapply(x,y,mean)

sds <- tapply(x,y,sd)

val <- pnorm(0,means[1]-means[2],sqrt(sds[1]^2+sds[2]^2))

vals <- c(val,1-val)}

if (norm == FALSE){

xy1 <- x[y==unique(y)[1]]

xy2 <- x[y==unique(y)[2]]

xy1_1 <- sample(xy1,reps,replace=TRUE)

xy2_1 <- sample(xy2,reps,replace=TRUE)

vals <- table(sign(xy2_1 - xy1_1))/reps}

return(vals)}

The function removes missing values if coded as NA (the standard for R) and checks to make sure that the second variable has only two values. It has options for the number of pairs to draw, the default being 10,000, but the function samples these together in two calls to the function sample, rather than in an explicit loop, so this number can be increased without much cost in time using the reps option. A smaller number could be useful if the function is to be bootstrapped. If the user is willing to assume that both samples are normally distributed, then norm=TRUE can be used. The following are example calls to the function:

> set.seed(305)

> y <- rbinom(1000,1,.4)

> x <- rnorm(1000,y,y+1)

> paircompare (x,y,reps=10000)

-1 1

0.6911 0.3089

> paircompare (x,y,norm=TRUE)

[1] 0.6746879 0.3253121

> y <- rbinom(1000,1,.4)

> x <- rbinom(1000,8,y/5+.5)

> paircompare (x,y,reps=10000)

-1 0 1

0.1505 0.1447 0.7048

> paircompare (x,y,norm=TRUE)

[1] 0.7819207 0.2180793
